# Serum Hepcidin Concentrations Decline during Pregnancy and May Identify Iron Deficiency: Analysis of a Longitudinal Pregnancy Cohort in The Gambia^1^,^2^,^3^

**DOI:** 10.3945/jn.116.245373

**Published:** 2017-04-19

**Authors:** Amat Bah, Sant-Rayn Pasricha, Momodou W Jallow, Ebrima A Sise, Rita Wegmuller, Andrew E Armitage, Hal Drakesmith, Sophie E Moore, Andrew M Prentice

**Affiliations:** 4 Medical Research Council (MRC) Unit The Gambia–MRC International Nutrition Group, Banjul, Gambia;; 5 MRC Human Immunology Unit, MRC Weatherall Institute of Molecular Medicine, University of Oxford, Oxford, United Kingdom;; 6 Division of Women’s Health, King’s College London, London, United Kingdom; and; 7 MRC Unit The Gambia–MRC International Nutrition Group and London School of Hygiene and Tropical Medicine, London, United Kingdom

**Keywords:** hepcidin, anemia, iron deficiency, pregnancy, diagnostic

## Abstract

**Background:** Antenatal anemia is a risk factor for adverse maternal and fetal outcomes and is prevalent in sub-Saharan Africa. Less than half of antenatal anemia is considered responsive to iron; identifying women in need of iron may help target interventions. Iron absorption is governed by the iron-regulatory hormone hepcidin.

**Objective:** We sought to characterize changes in hepcidin and its associations with indexes of iron stores, erythropoiesis, and inflammation at weeks 14, 20, and 30 of gestation and to assess hepcidin’s diagnostic potential as an index of iron deficiency.

**Methods:** We measured hemoglobin and serum hepcidin, ferritin, soluble transferrin receptor (sTfR), and C-reactive protein (CRP) at 14, 20, and 30 wk of gestation in a cohort of 395 Gambian women recruited to a randomized controlled trial. Associations with hepcidin were measured by using linear regression, and hepcidin’s diagnostic test accuracy [area under the receiver operating characteristic curve (AUC^ROC^), sensitivity, specificity, cutoffs] for iron deficiency at each time point was analyzed.

**Results:** The prevalence of anemia increased from 34.6% at 14 wk of gestation to 50.0% at 20 wk. Hepcidin concentrations declined between study enrollment and 20 wk, whereas ferritin declined between 20 and 30 wk of gestation. The variations in hepcidin explained by ferritin, sTfR, and CRP declined over pregnancy. The AUC^ROC^ values for hepcidin to detect iron deficiency (defined as ferritin <15 μg/L) were 0.86, 0.83, and 0.84 at 14, 20, and 30 wk, respectively. Hepcidin was superior to hemoglobin and sTfR as an indicator of iron deficiency.

**Conclusions:** In Gambian pregnant women, hepcidin appears to be a useful diagnostic test for iron deficiency and may enable the identification of cases for whom iron would be beneficial. Hepcidin suppression in the second trimester suggests a window for optimal timing for antenatal iron interventions. Hemoglobin does not effectively identify iron deficiency in pregnancy. This trial was registered at www.isrctn.com as ISRCTN49285450.

## Introduction

More than 38% of pregnant women worldwide are anemic, with the prevalence greatest in sub-Saharan Africa and parts of Asia (1). In Africa and Asia, anemia directly or indirectly contributes to one-quarter of maternal deaths (2). Consequences of antenatal anemia include premature delivery and low birth weight (3). Universal iron supplementation is recommended by the WHO for all pregnant women in a setting where anemia prevalence in this population exceeds 40% (4). However, only half of anemia cases in pregnancy worldwide (including only 44% of cases in Africa and 47% of cases in Asia) are attributed to iron deficiency and amenable to iron supplementation (1). The use of iron to treat other causes of anemia (including malaria, inflammation, and hemoglobinopathy) may not be beneficial, may represent a missed opportunity to identify and treat an alternative condition, and in some cases, may be actively harmful because it could exacerbate the risk of infection (5) or iron overload (6). However, such cases would likely be misdiagnosed and iron supplementation provided if a decision for iron intervention was based on a diagnosis of anemia, or if universal iron interventions are provided. Therefore, an appropriate case definition of iron deficiency in pregnancy could be of value in correctly identifying women who would most benefit from iron interventions.

Although there are several well-established tests for iron status, accurate assessment with the use of a single test is difficult (7, 8). Definitions of iron deficiency with the use of conventional markers, such as ferritin, remain under review (9) and may require adjustment for levels of inflammation. However, deploying multiple tests in the field is complex and costly, requires sophisticated interpretation, and is of limited value in resource-poor settings (7, 10). A biomarker that enables accurate diagnosis of iron status is needed.

Hepcidin, a peptide hormone produced by the liver, is the master regulator of systemic iron homeostasis (11). Hepcidin binds to the iron exporter ferroportin, inducing its internalization and subsequent degradation (12). Hepcidin concentrations are suppressed in iron deficiency, facilitating increased iron absorption and utilization, and elevated in iron loading and inflammation, preventing access of iron to the plasma. There is therefore considerable interest in pursuing hepcidin as a diagnostic test for iron status (13). We have previously found that hepcidin is a promising tool to identify individuals who might gain the most benefit from iron supplementation and defined putative thresholds that could help define iron deficiency in young children (14) and in women (15).

Small longitudinal studies showed that hepcidin is suppressed in pregnancy (16), likely facilitating the recognized increase in iron absorption seen during this period (17). Whether hepcidin suppression is mediated chiefly by iron deficiency (18) or maternal or fetal erythropoiesis, or another fetal, placental, or maternal factor, is currently unclear (16). Experimental data indicate that hepcidin may be directly transcriptionally regulated by estrogen and that steroid hormones may directly upregulate hepcidin expression (19, 20). The value of hepcidin as an index for iron deficiency in pregnancy has not been previously established, and putative cutoffs have not been defined. In a longitudinal cohort of pregnant Gambian women, we sought to evaluate associations between variables of maternal iron status and erythropoiesis with hepcidin at 3 distinct time periods and then to determine the diagnostic test accuracy and estimate potential cutoffs of hepcidin as an index of iron deficiency.

## Methods

### 

#### Participants and study design.

Samples were derived from the Early Nutrition and Immune Development (ENID)^9^ trial in rural Gambia, which is a randomized trial assessing whether nutritional supplementation to pregnant women and their infants can enhance infant immune development (trial registration: ISRCTN49285450) (21). For the main ENID trial, all of the women in the 36 villages who were registered within the West Kiang Demographic Surveillance System and aged between 18 and 45 y were invited from February 2010 to January 2013 to participate in the study. Women with confirmed pregnancy between 10 and 20 wk by ultrasound were randomly assigned to 1 of 4 intervention groups: *1*) iron-folic acid (standard care), *2*) multiple micronutrients (including iron-folic acid), *3*) protein energy plus iron-folic acid, and *4*) protein energy plus multiple micronutrients (including iron-folic acid). Women who were *1*) currently beyond 20 wk of gestation at the first clinic attendance (by ultrasound), *2*) enrolled in another study, *3*) severely anemic at recruitment (hemoglobin <7 g/dL), or *4*) menopausal were excluded. Samples from the first 400 women recruited to the ENID trial were included in this analysis. Thus, all of the participants in this substudy analysis received 60 mg Fe and 400 μg folic acid/d as part of the ENID intervention, from enrollment to delivery, as per current WHO guidelines (4, 21). The national prevalence of anemia in pregnancy was estimated at 67.9% (22). It would therefore not have been ethical to deny iron to women in pregnancy given the established benefits from iron supplementation.

#### Analytical methods.

Maternal blood samples collected at enrollment (booking) of mean gestational ages of 14, 20, and 30 wk of gestation were used for sample analysis. All of the samples were collected after an overnight fast, before 0900. Hemoglobin was analyzed from whole-blood samples by using a Medonic M-Series automated hematology analyzer (Boule Medical) shortly after sample collection. Serum ferritin, soluble transferrin receptor (sTfR), iron, and C-reactive protein (CRP) were analyzed from serum samples by using an automated biochemistry analyzer (COBAS Integra 400 plus; Roche Diagnostics). Serum hepcidin was quantified on the same samples by using a competitive ELISA (Bachem Hepcidin-25; now marketed by Peninsula Laboratories International), with a detection range of 0.049–25 ng/mL. Concentrations were interpolated from a 4-parameter curve fitted from a 2-fold, 10-point serial dilution made from a manufacturer-provided standard peptide. Samples outside the standard curve were re-analyzed at a higher dilution, and the final concentration was calculated on the basis of the dilution factor (23). The concentrations were obtained from the standard curve by using Dynex Revelation software (Dynex Technologies). Hepcidin measurements were performed in duplicate. All of the analyses were performed in the MRC (Medical Research Council) Keneba Laboratory. Results of conventional iron indexes were not available to staff measuring hepcidin, and vice versa.

#### Statistical analysis.

Variables were summarized at each time point, and means or proportions between time points compared by using *t* tests or 2-sample tests of proportions (2-sided, α = 0.05; significance defined as *P* < 0.05). Next, we modeled determinants of hepcidin concentrations at each of the 3 time points with the use of multiple linear regression, with variables log-transformed if the distribution was skewed. We estimated β-coefficients (which normalize the mean and SD), enabling comparison of associations between variables. We then generated nonparametric receiver operating characteristic curves (ROCs) and calculated the AUC^ROC^ together with Bamber and Hanley CIs for hepcidin concentration as a test of iron deficiency as defined by 2 recognized reference standards: *1*) serum ferritin <15 μg/L (24) and *2*) estimated body iron stores <0 mg/kg (based on the ratio of sTfR and ferritin) (10). The sensitivity and specificity of hepcidin as an index of iron deficiency were determined for each possible cutoff for hepcidin. We calculated the Youden index [(sensitivity/100 + specificity/100) − 1] at each value of hepcidin to assist in the selection of an optimal cutoff. Missing data were analyzed by list-wise deletion. The sample size of the study was predicated on the size of the cohort. Analyses were undertaken by using Stata 13 (StataCorp).

#### Ethics.

Informed consent (including permission to undertake future related analysis of the samples) was obtained from all participants through either a signature or a thumbprint. The ENID trial was approved by the Gambia Government/MRC Unit The Gambia joint ethics committee (SCC1126v2).

## Results

Samples from a total of 395 pregnant women were analyzed at recruitment (∼14 wk of gestation) and at 20 and 30 wk of gestation (**Table 1**). The mean age of the women was 29.6 y (95% CI: 29.1, 30.3), mean weight was 54.9 kg (95% CI: 53.9, 55.9 kg), mean height was 161.4 cm (95% CI: 160.9, 162.1 cm), and mean BMI (in kg/m^2^) was 21.0 (95% CI: 20.6, 21.3). Hematologic and iron indexes at each time point are shown in Table 1. Hepcidin concentrations were lower at 20 wk than at 14 wk and even lower at 30 wk of gestation. Hemoglobin declined from 14 wk through 30 wk of gestation, whereas serum ferritin decreased, sTfR increased, and hence total body iron declined, between 20 and 30 wk of gestation. CRP increased significantly between 14 and 20 wk of gestation.

**TABLE 1:** Iron and hematologic indexes in pregnant Gambian women^1^

Index	14 wk	20 wk	*P*	30 wk	*P*
*n*	395	375		367	
Gestational age, wk	14.1 (8.0, 21.3)	20.4 (15.0, 26.9)		30.5 (25.4, 34.4)	
Hemoglobin, g/dL	11.55 (7.2, 17.9)	11.00 (7.4, 14.5)	<0.001	10.77 (6.2, 14.4)	<0.0001
MCV, fL	81.9 (60.9, 98.6)	83.8 (62.9, 104.0)	<0.001	83.6 (61.8, 99)	NS
Serum ferritin, μg/L	20.69 (0.1, 237.2)	19.20 (0.1, 273.8)	NS	14.29 (0.1, 315.5)	<0.001
Serum sTfR, mg/L	4.41 (0.58, 17.97)	4.25 (1.12, 15.42)	NS	4.80 (1.49, 17.81)	<0.001
Serum sTfR-F index	3.29 (−14.80, 111.63)	3.26 (−5.63, 56.43)	NS	3.86 (−87.19, 76.26)	NS
Serum CRP, mg/L	1.70 (0.02, 43.48)	2.41 (0.00, 59.32)	0.001	2.29 (0.01, 126.68)	NS
Total body iron, mg/kg	2.70 (−18.38, 14.40)	2.50 (−17.57, 14.06)	NS	1.18 (−17.84, 11.02)	<0.001
Serum hepcidin, ng/L	1.59 (0.03, 49.79)	1.23 (0.02, 45.70)	0.006	1.09 (0.04, 135.69)	NS

1 Values are arithmetic (hemoglobin, MCV, sTfR, CRP, and total body iron) or geometric (ferritin, sTfR-F index, and hepcidin) means (ranges). *P* values are for 2-sided paired *t* tests comparing analytes between 14 and 20 wk and between 20 and 30 wk of gestation. CRP, C-reactive protein; MCV, mean cell volume; sTfR, soluble transferrin receptor; sTfR-F index, sTfR/log_10_ (ferritin).

The prevalence of anemia, iron deficiency, and inflammation across pregnancy is presented in **Table 2**. The prevalence of anemia was 34.6% at 14 wk and increased to 50.0% at 20 wk and remained stable thereafter. Iron deficiency defined as ferritin <15 μg/L was 37.6% at 14 wk and 34.3% at 20 wk, increasing to 50.6% at 30 wk of gestation. Iron deficiency defined as body iron <0 mg/kg was 27.35%, 24.78%, and 35.05% at 14, 20, and 30 wk of gestation, respectively. The prevalence of elevated sTfR likewise increased between 20 and 30 wk. The prevalences of iron deficiency anemia (anemia and low ferritin) were 18.8%, 20.16%, and 32.83% at 14, 20, and 30 wk of gestation, respectively. Thus, these data indicate that the prevalence of anemia increases during the second trimester of pregnancy, whereas the prevalence of iron deficiency increases between the second and early third trimester.

**TABLE 2:** Prevalence of anemia, iron deficiency, and inflammation at 14, 20, and 30 wk of gestation in pregnant Gambian women^1^

Condition	14 wk	20 wk	*P*	30 wk	*P*
Anemia, *n*/total *n*	135/390	189/378	<0.001	201/368	0.207
Hemoglobin <11 g/dL, % (95% CI)	34.62 (30.04, 39.50)	50.00 (44.95, 55.04)		54.62 (49.47, 59.66)	
Iron deficiency, *n*/total *n*	145/385	117/341	0.348	159/314	<0.0001
Ferritin <15 μg/L, % (95% CI)	37.66 (32.93, 42.63)	34.31 (29.43, 39.53)		50.64 (45.09, 56.16)	
IDA, *n*/total *n*	70/387	73/362	0.470	110/335	<0.001
Anemia + low ferritin, % (95% CI)	18.09 (14.54, 22.26)	20.16 (16.33, 24.64)		32.83 (27.99, 38.07)	
Elevated sTfR, *n*/total *n*	152/382	118/347	0.106	133/310	0.019
sTfR >4.4 mg/L, % (95% CI)	39.79 (34.97, 44.80)	34.01 (29.18, 39.17)		42.90 (37.47, 48.50)	
Absent body iron, *n*/total *n*	102/373	84/339	0.436	102/291	0.005
<0 mg/kg, % (95% CI)	27.35 (23.04, 32.11)	24.78 (20.45, 29.67)		35.05 (29.75, 40.74)	
Inflamed, *n*/total *n*	83/375	100/345	0.035	69/302	0.076
CRP >5 mg/L, % (95% CI)	22.13 (18.20, 26.63)	28.99 (24.41, 34.02)		22.85 (18.43, 27.95)	

1 *P* values are for 2-sided tests of proportion comparing proportions between 14 and 20 wk and between 20 and 30 wk of gestation. CRP, C-reactive protein; IDA, iron deficiency anemia; sTfR, soluble transferrin receptor; sTfR-F index, sTfR/log_10_ (ferritin).

Changes in concentrations of hepcidin and iron indexes at different gestation durations led us to evaluate the relation between hepcidin and these variables at each time point. Thus, we undertook multiple linear regression to compare associations between hepcidin at each of the 3 time points with 3 factors likely to influence it: ferritin (reflecting iron stores), sTfR (reflecting tissue iron demand including erythropoiesis), and CRP (reflecting inflammation). We used standardized coefficients to enable comparison in slope and the strength of association between each variable at each time point. As shown in **Table 3**, the slope of association between hepcidin and ferritin diminished over the course of pregnancy, whereas the association between hepcidin and sTfR was strengthened. Finally, the overall amount of variation in hepcidin explained by these variables diminished over the course of pregnancy.

**TABLE 3:** Factors associated with (log) hepcidin at 14, 20, and 30 wk of gestation in pregnant Gambian women by multiple regression^1^

	14 wk	*P*	20 wk	*P*	30 wk	*P*
Ferritin	0.60	<0.001	0.51	<0.001	0.42	<0.001
sTfR	−0.16	<0.001	−0.23	<0.001	−0.33	<0.001
CRP	0.04	0.327	0.02	0.696	0.07	0.139
Overall *r*^2^	0.48		0.40		0.39	

1 Values are β-coefficients (regression coefficients adjusted for a mean and SD of 1) and *P* values for association. CRP, C-reactive protein; sTfR, soluble transferrin receptor.

**FIGURE 1:**
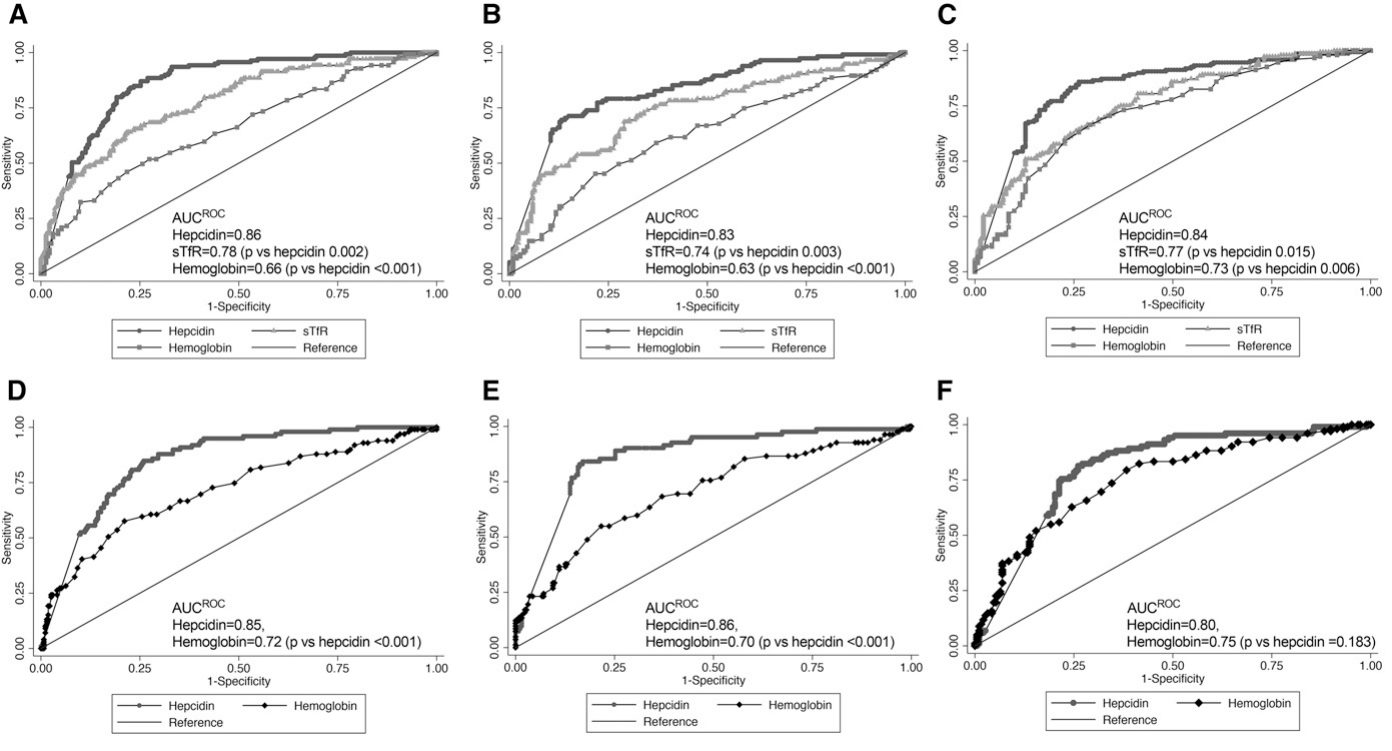
ROCs and the corresponding AUC^ROC^ values for hepcidin as a test for iron deficiency defined by 2 reference standards in pregnant Gambian women. (A–C) Capacity of hepcidin to detect iron deficiency as defined by ferritin <15 μg/L at 14 (A), 20 (B), and 30 wk (C) of gestation, respectively; the performance of hepcidin is compared with sTfR and hemoglobin. (D–F) Capacity of hepcidin to detect iron deficiency defined by body iron stores <0 mg/kg at 14 (D), 20 (E), and 30 wk (F) of gestation, respectively; the performance of hepcidin is compared with hemoglobin. ROC, receiver operating characteristic curve; sTfR, soluble transferrin receptor.

We then graphed the ROC curves and estimated AUC^ROC^ for hepcidin to detect iron deficiency. With the use of a standard definition of iron deficiency of ferritin <15 μg/L, the AUC^ROC^ values for hepcidin to detect iron deficiency were 0.86, 0.83, and 0.84 at 14, 20, and 30 wk, respectively. With the use of body iron <0 mg/kg, the AUC^ROC^ values for hepcidin to detect iron deficiency were 0.85, 0.86, and 0.80 at 14, 20, and 30 wk, respectively. During pregnancy, iron supplementation should be routinely administered when body iron stores and dietary iron cannot meet maternal, fetal, and placental demands (4, 25). However, hemoglobin remains the most commonly deployed initial test to determine the need for treatment doses of iron in pregnancy (26), even though it is a test for anemia rather than iron deficiency per se, because the conditions have often been considered synonymous (27). sTfR is another increasingly widely available index of iron status. We therefore compared the capacity of hepcidin with hemoglobin concentration and sTfR to detect iron deficiency (defined by low body iron stores) and found that hepcidin was superior to hemoglobin at 14 and 20 wk, and similar at 30 wk, and was superior to sTfR at each time point when using ferritin alone as a gold standard (**Figure 1**).

Sensitivity, specificity, and the Youden index for a range of potential hepcidin cutoffs are shown in **Table 4**. Hepcidin appears to have an optimal cutoff (based on the tradeoff between sensitivity and specificity calculated by the Youden index) that fluctuates over the duration of pregnancy (from 1.5–2.0 ng/mL at booking, to 0.5 ng/mL at 20 wk, to 1.0–1.5 ng/mL by 30 wk). However, these lower thresholds are associated with reductions in sensitivity. The prevalence of iron deficiency at each hepcidin threshold is presented in **Supplemental Table 1**.

**TABLE 4:** Sensitivity, specificity, and the Youden index at putative hepcidin thresholds at each time point in pregnancy in pregnant Gambian women^1^

	14 wk			20 wk			30 wk		
Hepcidin cutoff, ng/mL	Sensitivity	Specificity	Youden	Sensitivity	Specificity	Youden	Sensitivity	Specificity	Youden
Ferritin <15 μg/L as standard									
0.16	42.1	93.3	0.354	60	89.7	0.497	52.2	90.9	0.431
0.5	56.6	87.9	0.445	72.2	81.7	0.539	69.2	85.1	0.543
1.0	76.6	81.3	0.579	79.1	71.4	0.505	76.1	80.5	0.566
1.5	84.1	77.1	0.612	80	65.2	0.452	83.6	76	0.596
2.0	88.3	70.8	0.591	83.5	59.4	0.429	85.5	70.1	0.556
2.5	93.1	66.3	0.594	86.1	52.2	0.383	86.2	64.9	0.511
3.0	93.8	62.1	0.559	89.6	48.2	0.378	88.7	59.7	0.484
Total body iron <0 mg/kg as standard									
0.16	50	90.4	0.404	69.5	86.3	0.558	58.8	82	0.408
0.5	63.7	83.4	0.471	85.4	78.8	0.642	78.4	74.6	0.530
1.0	82.4	74.2	0.566	90.2	68.6	0.588	84.3	69.3	0.536
1.5	87.3	68.6	0.559	90.2	62.7	0.529	88.2	61.9	0.501
2.0	91.2	83.9	0.751	92.7	56.9	0.496	90.2	56.6	0.468
2.5	95.1	57.6	0.527	95.1	50.2	0.453	91.2	52.4	0.436
3.0	95.1	53.5	0.486	95.1	45.1	0.402	95.1	48.1	0.432

1 Sensitivity = true positive detection rate; specificity = true negative detection rate; Youden index = (sensitivity/100 + specificity/100) − 1 for each time point by using the 2 gold standards of iron deficiency considered.

## Discussion

In this longitudinal study in pregnant women, we measured hepcidin concentrations together with traditional iron biomarkers, investigated the changes that occurred between 14 and 30 wk of gestation, and assessed the diagnostic performance of hepcidin for iron deficiency. We observed that hepcidin concentrations decreased by 20 wk of gestation, whereas iron stores (measured by ferritin and body iron stores) declined most substantially at 30 wk of gestation. These changes occurred despite the distribution of routine iron supplementation to all of the women in the cohort. We observed that hepcidin performs well as a diagnostic test for iron deficiency, and indeed outperforms hemoglobin at all time points. On the basis of the AUC^ROC^, the diagnostic performance of hepcidin to detect iron deficiency was good (AUC^ROC^ >0.80) and was similar from 14 to 30 wk of gestation.

Few studies, to our knowledge, have previously reported on hepcidin concentrations during human pregnancy, and those that did generally included relatively small sample sizes. Van Santen et al. (28) measured hepcidin in 31 women across the 3 trimesters of pregnancy and observed that hepcidin concentrations decreased from the second trimester of pregnancy and became essentially undetectable by the third trimester; hepcidin concentrations correlated with iron status. A study in 37 Danish women receiving iron supplementation in pregnancy likewise found that hepcidin concentrations were suppressed during pregnancy, occurring between the first measurement at 13–20 wk and the second measurement at 21–28 wk; hepcidin concentrations were observed to remain suppressed during pregnancy and to increase at delivery and thereafter (29). In contrast, Simavli et al. (30) measured hepcidin concentrations across pregnancy in healthy Turkish women and observed no reduction in hepcidin across pregnancy, nor an association between hepcidin and iron status. Interestingly, in a similar study, the authors found evidence that elevated hepcidin in the second trimester may be associated with adverse pregnancy outcomes, such as pre-eclampsia and intrauterine growth retardation (31). The importance of hepcidin in facilitating increased iron absorption was confirmed in a stable isotope iron study that showed a correlation between maternal hepcidin concentrations and maternal iron absorption and transfer of iron to the neonate (32).

By studying hepcidin concentrations across gestation in a large cohort of women with uncomplicated singleton pregnancies, all of whom were randomly assigned to receive iron supplementation, in a population at high risk of anemia, we confirm that hepcidin concentrations decline by 20 wk of pregnancy, before the onset of biochemical evidence of iron deficiency or clear evidence of changes in iron stores (assessed by ferritin, sTfR, and total body iron). Conversely, the reduction in iron stores observed between 20 and 30 wk of pregnancy was not accompanied by a further reduction in hepcidin concentrations. At all times in pregnancy, hepcidin is associated with ferritin, sTfR, and CRP, but the proportion of variation in hepcidin attributable to these factors decreases during gestation. These findings suggest that iron deficiency itself is not solely responsible for the reduction in hepcidin during pregnancy and raise the possibility of an additional, as yet unidentified, regulator of hepcidin concentrations during pregnancy. The expansion of maternal erythropoiesis with the expression of the erythroid-derived hepcidin-suppression hormone erythroferrone represents one hypothesis (33), which is supported by the increasing regression coefficient between hepcidin and sTfR (an indicator of RBC production) as pregnancy progresses. Alternatively, an as-yet-undiscovered placenta- or fetus-derived factor may act to suppress hepcidin expression in the maternal liver. Estrogen and progesterone appear to upregulate hepcidin transcription in cellular and animal models (20), but their role in regulating iron homeostasis in human pregnancy requires further investigation. The expansion of plasma volume during pregnancy causes reductions in concentrations of many analytes, including hemoglobin (hemodilution), and has been considered a potential mechanism for reductions in concentrations of ferritin (34). However, such a mechanism would not explain concordant increases in concentrations of sTfR, suggesting that the changes in measured iron status are related to changes in iron stores and metabolism, rather than exclusively to hemodilution.

The suppression of serum hepcidin is an essential part of the physiologic response to iron need, and pregnant women with undetected concentrations of serum hepcidin transfer dietary iron to their fetus more effectively (32). As the direct regulator of systemic iron homeostasis through its role in governing cellular iron export, and hence absorption across the enterocyte (12), hepcidin is an intriguing candidate as a potential guide to determine which individuals should be considered for iron interventions. Our data indicate a hepcidin threshold with the use of this assay that fluctuates over pregnancy, from between 1.5 and 2.0 ng/mL at 14 wk to lower thresholds at 30 wk of gestation; reductions in these cutoffs are associated with improved specificity but come at the necessary expense of sensitivity. Importantly, reductions in hepcidin concentrations in pregnancy reflect a physiologic state of high iron requirement. Thus, although low hepcidin concentrations likely appropriately identify individuals in whom iron supplementation may be beneficial, it is unclear whether depriving women of iron supplements on the basis of hepcidin concentrations that are low but above the threshold we identified is beneficial. Given the established benefits from iron supplements on maternal and child health and the paucity of data indicating that iron is harmful in this group (35), it may be reasonable to use a higher hepcidin threshold to identify women in whom iron should be withheld, which is more “sensitive.” This would need to be confirmed in prospective studies. Our data indicate that hepcidin generally outperforms hemoglobin measurement as an index of iron deficiency, reinforcing the concept that hemoglobin testing alone is not an adequate approach to determining iron stores and the need for iron interventions. As we showed previously in children (14), testing for anemia is an inaccurate approach for the detection of iron deficiency. However, in both high-income (36) and low-income (37) settings, individual decisions to treat iron deficiency are routinely based on hemoglobin, rather than on iron deficiency testing. In population health, the dose of iron for the universal distribution of iron interventions is based on the prevalence of anemia, not iron deficiency (4). Our data suggest that measurement of an iron variable (including hepcidin) may better guide individual or public health approaches. Although rural Gambia is a malaria-endemic region, recent data from both the health center and community surveys showed that malaria endemicity in The Gambia is now low, heterogeneous, and seasonal (38), and malaria did not affect this cohort. Approximately one-fifth of the cohort had inflammation as defined by CRP >5 mg/L. CRP did not correlate with hepcidin in this population, indicating that inflammation was not an important regulator of hepcidin in these women. However, CRP-based definitions of inflammation are indistinct in pregnancy because CRP is elevated over the course of gestation (39) and may hence be an imperfect biomarker for inflammation in this context.

To our knowledge, the diagnostic performance of hepcidin to detect iron deficiency has not been previously evaluated in pregnancy. However, studies in nonpregnant women found hepcidin to be a promising indicator of iron deficiency. When hepcidin was compared with the sTfR/log_10_ (ferritin) index as a standard, hepcidin showed an AUC^ROC^ of 0.89, and when compared with ferritin, it showed an AUC^ROC^ of 0.87 (15). With the use of the same hepcidin ELISA kit as the current study (Bachem), we assessed the diagnostic performance of hepcidin as an index of iron deficiency and the need for iron supplementation in West and East African preschool children and found that the AUC^ROC^ for hepcidin to identify iron deficiency was 0.85, with a threshold that used the Bachem ELISA of 5.5 ng/mL to distinguish iron deficiency across the overall population and among anemic children (14). Given the suppression of hepcidin concentrations in pregnancy, it is unsurprising that a lower optimal threshold would be identified in this population. There are several assays available for hepcidin measurement, which use ELISA or MS methodology. Hepcidin concentrations measured by these different assays are correlated but differ in absolute values (40). If hepcidin assays could be harmonized, the thresholds identified by our study may serve as a platform for a value that could be used to detect iron deficiency in pregnancy. A key advantage of hepcidin measurement is that it directly interrogates systemic iron handling, and hence predicts absorption and utilization of ingested iron (41). Given that hepcidin transcription is directly regulated by iron stores, erythropoiesis, and inflammation (42), hepcidin measurements represent the net integration of these signals, which currently have to be measured by individual biomarkers [e.g., ferritin, hemoglobin (or sTfR), and CRP or α1-glycoprotein, respectively]. Thus, a single biomarker may be able to replace multiple indexes, which may obviate the costs associated with the more current, relatively expensive assays.

Our study compares hepcidin with established reference standards frequently recommended for the diagnosis of iron deficiency in pregnancy. However, the optimal ferritin thresholds used to define iron deficiency remain uncertain and continue to be reviewed (9). Validation of iron deficiency in a large field study with gold-standard assessments, such as measurement of bone marrow iron stores or stable isotope iron incorporation, would not have been achievable, and hence our analysis represents the most pragmatic approach. Factors beyond changes in body iron stores, such as plasma dilution, could potentially explain reductions in ferritin over the course of pregnancy; however, these effects are unlikely to explain the changes in both hepcidin and ferritin because reductions in the biomarkers were seen at different time intervals (whereas hemoglobin declined constantly from 14 to 30 wk). Changes in transferrin saturation may acutely modulate hepcidin expression, but we were unable to include these data in this study (43).

An improved understanding of the complex and distinctive mechanisms of regulation of iron absorption, utilization, and transfer during pregnancy will enable improved targeting of clinical and public health interventions. Our data show the suppression of hepcidin concentrations among pregnant rural women by 20 wk of gestation, in advance of the onset of low iron stores, and suggest a window at the commencement of the second trimester before iron stores have declined when iron utilization may be greatest. In this population, the commonly used approach of identifying participants in need of iron treatment by screening for anemia (by measuring hemoglobin) is only modestly accurate. Associations between hepcidin and iron stores are maintained, although modified, across pregnancy and are reflected by the capacity of hepcidin to distinguish individuals in the population with iron deficiency. An approach for hepcidin-directed iron supplementation in both pregnant women and children is currently being tested in the field (44, 45).

## Footnotes

9 Abbreviations used: CRP, C-reactive Protein; ENID, Early Nutrition and Immune Development; IDA, iron deficiency anemia; ROC, receiver operating characteristic curve; sTfR, soluble transferrin receptor.
